# Sensing iron availability *via* the fragile [4Fe–4S] cluster of the bacterial transcriptional repressor RirA†
†Electronic supplementary information (ESI) available. See DOI: 10.1039/c7sc02801f


**DOI:** 10.1039/c7sc02801f

**Published:** 2017-10-23

**Authors:** Ma Teresa Pellicer Martinez, Ana Bermejo Martinez, Jason C. Crack, John D. Holmes, Dimitri A. Svistunenko, Andrew W. B. Johnston, Myles R. Cheesman, Jonathan D. Todd, Nick E. Le Brun

**Affiliations:** a Centre for Molecular and Structural Biochemistry , School of Chemistry , University of East Anglia , Norwich Research Park , Norwich , NR4 7TJ , UK . Email: n.le-brun@uea.ac.uk ; Fax: +44 1603 592003 ; Tel: +44 1603 592699; b School of Biological Sciences , University of East Anglia , Norwich Research Park , Norwich , NR4 7TJ , UK; c School of Biological Sciences , University of Essex , Wivenhoe Park , Colchester CO4 3SQ , UK

## Abstract

Rhizobial iron regulator A (RirA) is a global regulator of iron homeostasis in many nitrogen-fixing Rhizobia and related species of α-proteobacteria. It belongs to the widespread Rrf2 super-family of transcriptional regulators and features three conserved Cys residues that characterise the binding of an iron–sulfur cluster in other Rrf2 family regulators. Here we report biophysical studies demonstrating that RirA contains a [4Fe–4S] cluster, and that this form of the protein binds RirA-regulated DNA, consistent with its function as a repressor of expression of many genes involved in iron uptake. Under low iron conditions, [4Fe–4S] RirA undergoes a cluster conversion reaction resulting in a [2Fe–2S] form, which exhibits much lower affinity for DNA. Under prolonged low iron conditions, the [2Fe–2S] cluster degrades to apo-RirA, which does not bind DNA and can no longer function as a repressor of the cell's iron-uptake machinery. [4Fe–4S] RirA was also found to be sensitive to O_2_, suggesting that both iron and O_2_ are important signals for iron metabolism. Consistent with this, *in vivo* data showed that expression of RirA-regulated genes is also affected by O_2_. These data lead us to propose a novel regulatory model for iron homeostasis, in which RirA senses iron *via* the incorporation of a fragile iron–sulfur cluster that is sensitive to iron and O_2_ concentrations.

## Abstract 2

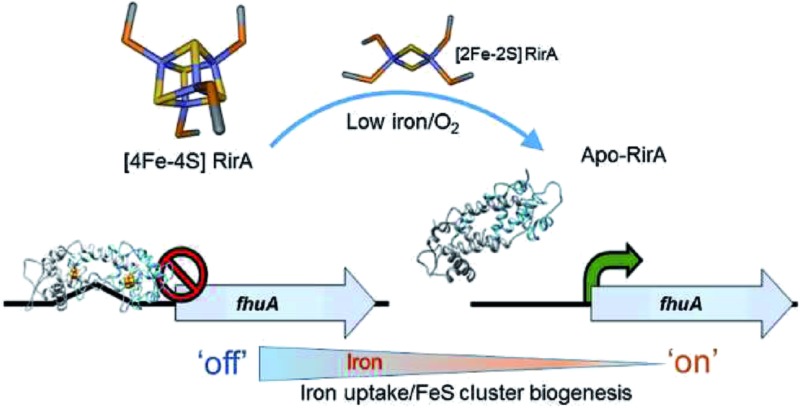
The global iron regulator RirA controls transcription of iron metabolism genes *via* the binding of a fragile [4Fe–4S] cluster.

## Introduction

Iron is essential for virtually all forms of life, but the redox properties that make it indispensable also mean that it is potentially extremely toxic. Thus, its concentration and speciation in the cell must be carefully controlled.^1^ A major part of cellular iron metabolism is the regulation of iron uptake and storage in response to iron availability. In the model organisms *Escherichia coli* and *Bacillus subtilis*, as well as in many other taxonomically diverse bacteria, this is achieved by the global iron regulator Fur (Ferric uptake regulator), which senses iron by binding Fe^2+^, in which form Fur binds to *cis*-acting “Fur boxes” near the promoters of genes involved in the iron-uptake machinery.^2^,^3^ In the Gram-positive bacterium *Corynebacterium diphtheriae* and related species, the iron regulator is DtxR (Diphtheria toxin Repressor), which is unrelated to Fur in terms of sequence but shares some structural features, and also binds Fe^2+^ as a co-repressor.^4^,^5^

In contrast, in some bacteria, a very different type of “global” iron-responsive regulator, termed RirA (Rhizobial iron regulator A),^6^–^14^ serves analogous functions as Fur/DtxR, but has no structural or sequence similarity to them. Discovered first in *Rhizobium leguminosarum*, the nitrogen-fixing symbiont that induces root nodules on peas, beans and clovers, RirA was shown to repress many genes involved in iron homeostasis, by binding to operator sequences known as “IRO boxes”.^8^ The RirA regulon includes genes for the synthesis (*vbs*) and uptake (*fhu*) of the siderophore vicibactin, genes involved in heme uptake (*hmu* and *tonB*), genes for the synthesis of iron–sulfur clusters (*suf*), the *irrA* regulatory gene (see below), as well as *rirA* itself.^6^–^9^ RirA also occurs in several closely related genera of α-proteobacteria, including other Rhizobia (*Mesorhizobium*, *Sinorhizobium*/*Ensifer*) and the pathogens *Bartonella*, *Brucella*, and *Agrobacterium* in which its regulatory properties have also been demonstrated,^12^,^13^,^15^ and RirA homologues exist in other α-proteobacteria isolated from a wide range of different environments (*e.g.*, *Martelella*, *Ochrobactrum*, *Shinella*).

**Fig. 1:**
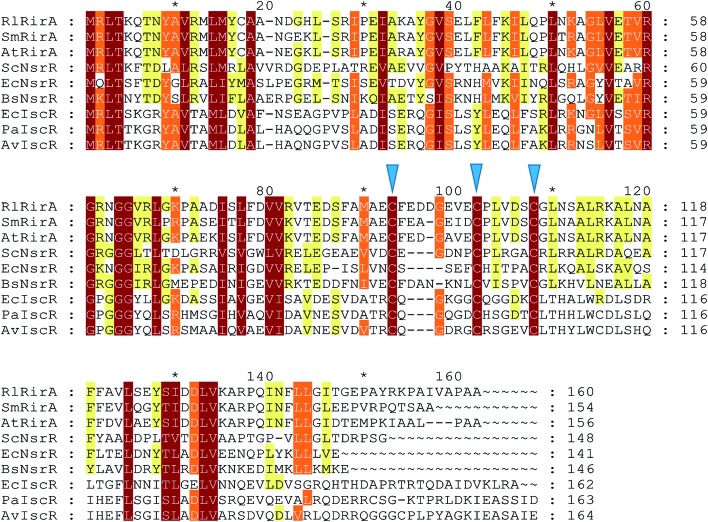
Alignment of *R. leguminosarum* RirA with other Rrf2 family regulators. Alignment of *R. leguminosarum* RirA (RlRirA, Accession number CAC35510.1) with RirA sequences from *Sinorhizobium*/*Ensifer meliloti* (SmRirA, Accession number WP_003527122) and *Agrobacterium tumefaciens* (AtRirA, Accession number WP_003514531), NsrR sequences from *Streptomyces coelicolor* (ScNsrR, Accession number WP_011031657), *Escherichia coli* (EcNsrR, Accession number WP_032251176) and *Bacillus subtilis* (BsNsrR, Accession number WP_063334953), and IscR sequences from *E. coli* (EcIscR, Accession number WP_053285796), *Pseudomonas aeruginosa* (PaIscR, Accession number WP_034033784) and *Azotobacter vinelandii* (AvIscR, Accession number WP_012702552). The three conserved cysteine residues predicted to ligate iron–sulfur clusters in Rrf2 family regulators are indicated by blue arrow heads. The alignment was carried out using Clustal Omega^67^ and Genedoc.^68^

RirA's lack of any sequence similarity to Fur or DtxR indicates that it is a novel type of iron responsive regulator. It is a member of the Rrf2 super-family of transcriptional regulators^16^ that includes IscR (regulator of iron–sulfur cluster biosynthesis)^17^,^18^ and NsrR (regulator of nitrosative stress response),^19^–^21^ both of which have been shown to bind an iron–sulfur cluster. Structures of apo-IscR and [4Fe–4S] NsrR,^17^,^21^ along with that of CymR,^22^ revealed a conserved elongated fold consisting of a largely α-helical structure with two anti-parallel β-strands comprising a DNA-binding domain (α_1_, α_2_, α_3_, β_1_, β_2_, α_4_) and a dimerization helix (α_6_, α_7_). The DNA-binding domain, which contains a winged helix-turn-helix motif, is connected to the dimerisation helix *via* a loop containing three Cys residues that are conserved in IscR and NsrR. The effects of substituting these with non-coordinating residues were consistent with a role in cluster coordination, and this was demonstrated in the NsrR structure.^21^,^23^–^25^ These Cys residues are also conserved in RirA (Fig. 1), suggesting that RirA may also be an iron–sulfur cluster-binding protein; indeed, substituting these residues with Ala in RirA of *A. tumefaciens* abolished its regulatory activity.^15^

In some Gram-negative α-proteobacteria, RirA functions alongside a second global iron regulator, Irr (Iron responsive repressor). This Fur-family protein has been shown to sense iron indirectly through the binding of heme.^26^–^28^ This raises the possibility that cellular iron regulation in Rhizobia occurs *via* two unusual global regulators, one that senses iron *via* iron–sulfur clusters and the other that operates *via* heme.

Here we report studies of *R. leguminosarum* RirA, using *in vitro* and *in vivo* approaches. The data demonstrate that RirA can bind a [4Fe–4S] cluster, and that this form of the protein binds to an IRO box sequence. Exposure to low iron conditions initiates loss of iron to generate a [2Fe–2S] form, which exhibits much weaker DNA-binding affinity. The [2Fe–2S] form is also unstable under prolonged low iron conditions, resulting in apo-RirA, which does not bind the IRO box sequence. [4Fe–4S] RirA is also sensitive to O_2_, leading us to propose a novel mechanism of iron sensing by RirA in which iron and O_2_ signals are integrated.

## Results and discussion

### Iron–sulfur cluster bound forms of RirA

**Fig. 2:**
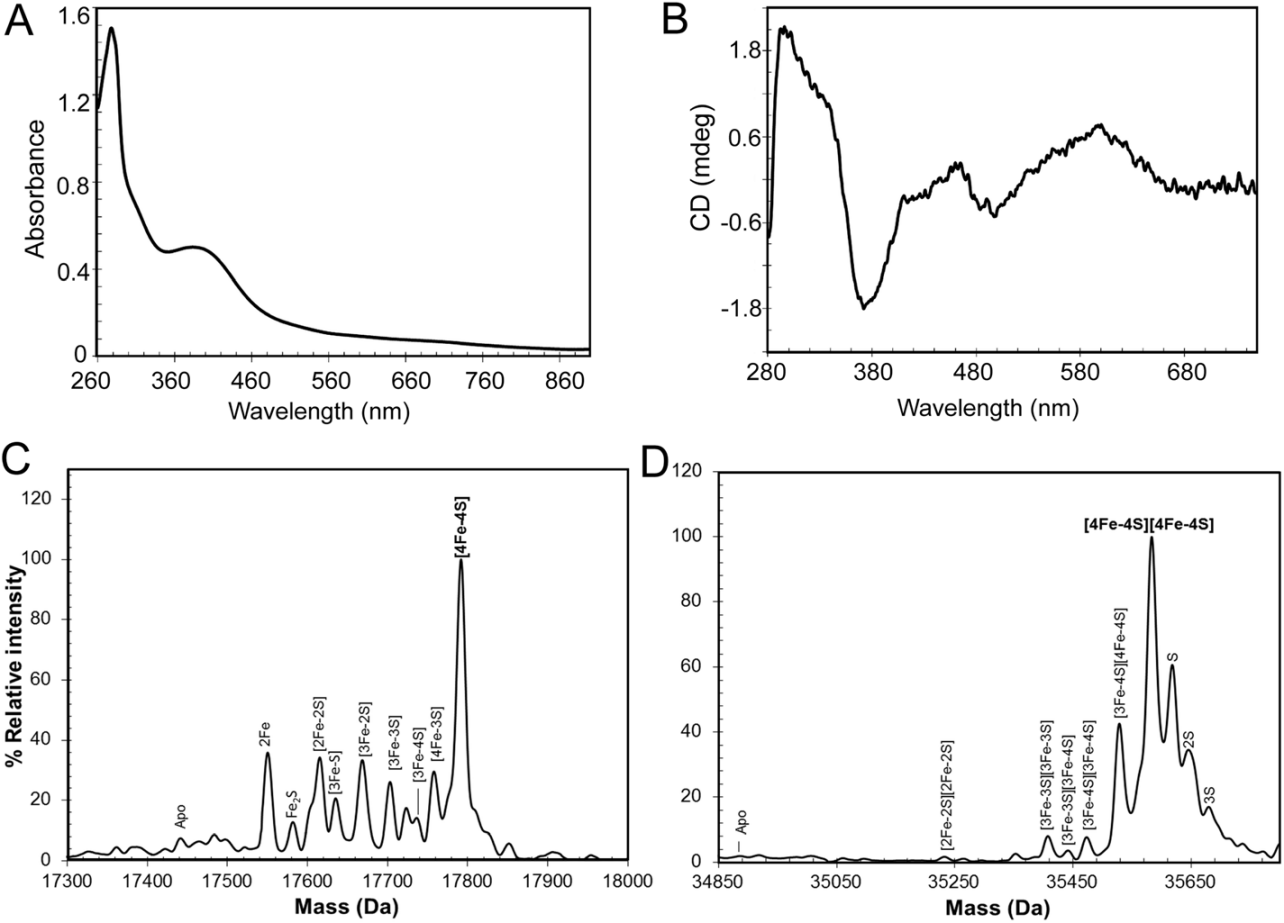
Characterization of the RirA iron–sulfur cluster. (A) UV-visible absorbance and (B) CD spectra of reconstituted RirA (371 μM [4Fe–4S] RirA) in buffer B. Pathlength 1 mm. (C) and (D) Positive ion mode ESI-MS deconvoluted spectra of RirA (30 μM in cluster in 250 mM ammonium acetate pH 7.32) under non-denaturing conditions in the monomer (C) and dimer (D) mass regions.

Purification of RirA following over-expression in *E. coli* resulted in a straw-brown colored solution. Iron and protein analyses revealed a low ratio of iron to protein (∼0.45 : 1), indicating that the majority of the protein was in the apo-form. A small proportion (<5%) of the protein was truncated (Fig. S1†), with the principal form lacking 20 C-terminal residues compared to the full length protein, as shown by mass spectrometry. The UV-visible spectrum (Fig. S1†) was also consistent with a very low cluster content (<10%). The broad absorbance across the near UV and visible regions suggested that, in the fraction of RirA molecules that did contain clusters, these were a mixture of both [2Fe–2S] and [4Fe–4S] forms (see below).^20^,^29^ *In vitro* cluster reconstitution of as isolated RirA resulted in a much darker brown solution, consistent with iron, sulfide and protein analyses, which showed that the protein could be loaded to give a maximum of 3.8 ± 0.27 iron and 3.6 ± 0.21 sulfide per protein, respectively. UV-visible absorbance and CD spectra of reconstituted RirA are shown in Fig. 2A and B. The form of the absorbance spectrum is characteristic of a [4Fe–4S] cluster, and while a wavelength maximum below 400 nm is unusual, several examples of this are known.^30^–^32^ Indeed, the CD spectrum is similar to that reported for [4Fe–4S] NsrR, further supporting the presence of a [4Fe–4S] cluster in RirA.^20^

Electrospray ionization mass spectrometry (ESI-MS) under non-denaturing conditions, where the cluster remains bound to the protein, has been shown recently to be a valuable technique for the identification of cluster type in a range of iron–sulfur regulatory proteins.^20^,^21^,^33^–^35^ Conditions were established for the ionization of cluster-reconstituted RirA under non-denaturing conditions. The *m*/*z* spectrum (Fig. S2†) contained peaks in two distinct regions, corresponding to monomeric RirA (600–1500 *m*/*z*), and dimeric RirA (1800–3000 *m*/*z*). The observation of dimeric RirA is consistent with analytical gel filtration (Fig. S3†) showing that RirA eluted at a volume that indicated a molecular mass of ∼39 kDa, while the calculated mass of monomeric RirA is 17.4 kDa. This is also consistent with all Rrf2 family regulators characterized to date being homodimers.^17^,^20^,^21^ The observation of monomeric RirA in the mass spectrum indicates that the protein monomerizes to a significant extent during ionisation, as recently observed for other dimeric iron–sulfur cluster regulators such as NsrR, RsrR and FNR.^21^,^33^,^34^

The major peak in the deconvoluted mass spectrum of RirA in the monomer region, Fig. 2C, was at 17 792 Da, corresponding to [4Fe–4S] RirA (see Table 1 for observed and predicted masses for RirA containing different iron–sulfur clusters). To the lower mass side of this major peak was a collection of smaller peaks corresponding to a range of cluster breakdown species, including [4Fe–3S], [3Fe–4S], [3Fe–3S], [3Fe–2S], [2Fe–3S], [2Fe–2S], as well as sodium adducts of [Fe–2S] and [Fe–S], see Fig. 2C and Table 1. Also, a small peak due to apo-protein was observed at 17 441 Da.

**Table 1:** Predicted and observed masses for apo- and cluster-bound forms of RirA

RirA species	Predicted mass^*a*^ (Da)	Observed mass^*b*^ (Da)	ΔMass^*c*^ (Da)
**Monomeric**			
Apo	17 442	17 441	–1
[4Fe–4S]^2+^	17 792	17 792	0
[4Fe–3S]^4+^	17 758	17 758	0
[3Fe–4S]^1+^	17 737	17 736	–1
[3Fe–3S]^3+^	17 703	17 703	0
[3Fe–2S]^5+^	17 669	17 668	–1
[3Fe–S]^7+^	17 635	17 635	0
[2Fe–2S]^2+^	17 616	17 615	–1
[2Fe–S]^4+^	17 582	17 582	0
2Fe^2+^	17 550	17 550	0

**Dimeric**			
Apo	34 884	—	—
[4Fe–4S]^2+^/[4Fe–4S]^2+^	35 584	35 585	–1
[3Fe–4S]^1+^/[4Fe–4S]^2+^	35 529	35 528	1
[3Fe–4S]^1+^/[3Fe–4S]^1+^	35 474	35 475	–1
[3Fe–3S]^3+^/[3Fe–4S]^1+^	35 440	35 441	–1
[3Fe–3S]^3+^/[3Fe–3S]^3+^	35 406	35 407	–1
[2Fe–2S]^2+^/[2Fe–2S]^2+^	35 232	35 232	0

^*a*^The predicted mass depends on the cluster/cluster fragment charge because binding is assumed to be charge compensated.^35^,^58^ Cluster charge states are as observed previously.^35^

^*b*^The average observed mass is derived from at least four independent experiments, with standard deviation of ±1 Da.

^*c*^The difference between the average observed and predicted masses.

The deconvoluted mass spectrum of the dimer region, Fig. 2D, contained a major peak at 35 583 Da, corresponding to the RirA dimer containing two [4Fe–4S] clusters (Table 1). To the higher mass side were three less abundant peaks at +32, +64 and +96 Da, corresponding to one, two and three sulfane sulfur adducts, which arise because Cys residues readily pick up additional sulfurs as persulfides, which, in some cases at least, can coordinate an iron–sulfur cluster.^33^,^36^ To the lower mass side were smaller peaks due to the RirA dimer containing [3Fe–4S]/[4Fe–4S], [3Fe–4S]/[3Fe–4S], and [3Fe–3S]/[3Fe–3S] clusters, with a very low intensity peak due to [2Fe–2S]/[2Fe–2S] RirA. Each of these most likely represents a breakdown product of one or both of the [4Fe–4S] clusters in the dimer. The observation of a range of cluster breakdown species is consistent with the fragility of the [4Fe–4S] cluster, though we note that there are considerably fewer breakdown species compared to the RirA monomer region. This could indicate that dissociation of the dimer into monomers results in an increased propensity for cluster breakdown during the MS experiment.

RirA belongs to a sub-set of Rrf2 proteins that contain three conserved Cys residues that are associated with coordination of an iron–sulfur cluster.^23^–^25^ Other members of this subset include IscR, the regulator of iron–sulfur biogenesis,^18^ and NsrR, the regulator of nitrosative stress response.^20^,^21^ Although both IscR and NsrR contain iron–sulfur clusters, these are of different types; IscR binds a [2Fe–2S] cluster^18^ and NsrR a [4Fe–4S] cluster.^20^,^21^ Thus, while the presence of these Cys residues and the effects of their substitution^15^ strongly suggested that RirA also is an iron–sulfur cluster regulator, the type of cluster was unknown. Here, we have established that, like NsrR, RirA can bind a [4Fe–4S] cluster, but the identity of the presumed fourth ligand of the RirA [4Fe–4S] cluster remains unknown. In [2Fe–2S] IscR, the fourth ligand is a His residue (His106 *E. coli* IscR numbering),^37^ while in [4Fe–4S] NsrR it is an Asp residue (Asp8), but neither of these residues is conserved in RirA (Fig. 1).^20^,^21^ We note that RirA contains a fourth Cys residue, located near its N-terminus, that could serve as the fourth ligand. An alignment suggests that this residue is conserved among RirA proteins but not in other Rrf2 family members, see Fig. S4.† There is clearly significant variability in the nature of the cluster coordination between members of the Rrf2 super-family, which is likely to be important in determining the type of iron–sulfur cluster that is bound and in tuning its functional properties.

### The [4Fe–4S] cluster of RirA is sensitive to O_2_

**Fig. 3:**
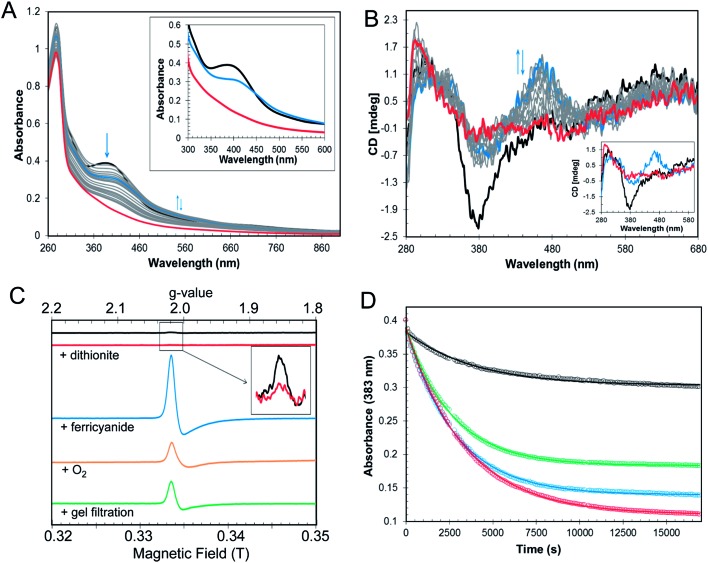
O_2_ sensitivity of [4Fe4S] RirA. (A) UV-visible absorption spectra and (B) CD spectra were recorded over several hours following exposure of [4Fe–4S] RirA (30 μM in cluster in buffer B) to 230 μM O_2_. Inset in (A) are absorbance spectra recorded at 0 min (black), 44 min (blue) and 24 h (1440 min, red). Inset in (B) are CD spectra recorded at 0 min (black), 44 min (blue) and 5.5 h (339 min, red). (C) EPR spectra of [4Fe–4S] RirA (black), and following: addition of 100 μM of sodium dithionite (red); addition of 100 μM of potassium ferricyanide (blue); exposure to O_2_ (orange); and, gel filtration (green). Inset is an expanded view of the signal in the reconstituted sample before and after addition of dithionite. RirA (100 μM [4Fe–4S] RirA) was in buffer B. (D) Plots of UV-visible absorbance at 383 nm as a function of time following addition of increasing concentrations of O_2_ to [4Fe–4S] RirA (30 μM in cluster). 58 μM O_2_ (black), 115 μM O_2_ (green), 172 μM O_2_ (blue) and 230 μM O_2_ (red). Fits to the data are represented by solid lines. Pathlength for all measurements was 1 cm.

In the symbiotic bacteroids of leguminous plant root nodules, O_2_ levels are kept sufficiently low to prevent damage to the N_2_-fixing nitrogenase enzyme, while permitting aerobic respiration.^38^ Under these conditions, it is unlikely that RirA would be exposed to very much O_2_. However, free-living *R. leguminosarum* (and many other RirA-containing α-proteobacteria) grow aerobically in the soil and will experience varying concentrations of O_2_, depending on conditions. Therefore, the sensitivity of [4Fe–4S] RirA to O_2_ was examined. Fig. S5† shows that, under anaerobic conditions, [4Fe–4S] RirA was entirely stable over a period of 7 h, and in fact could be stored for several weeks at 4 °C without degradation. UV-visible and CD spectra of [4Fe–4S] RirA in the presence of a saturating concentration of O_2_ (230 μM) revealed a pronounced red shift of the absorbance band at 383 nm over the first 45 min, followed by gradual loss of absorbance intensity (Fig. 3A). Changes in the CD spectrum were consistent with this, with an increase in the positive band at 464 nm over the first 45 min, followed by the loss of all bands as the apo-protein was formed (Fig. 3B). The observed changes are characteristic of the formation of a [2Fe–2S] cluster prior to complete loss of the cluster.

Conversion of [4Fe–4S] RirA to a [2Fe–2S] form could involve formation of a transiently stable [3Fe–4S]^1+^, which can be detected by electron paramagnetic resonance (EPR) spectroscopy.^29^ The EPR spectrum of reconstituted RirA (Fig. 3C) contained a very low intensity signal with *g*-value of 2.01, which is characteristic of a *S* = ½[3Fe–4S]^1+^ cluster.^39^ Quantification of the trace signal revealed that this accounted for <0.5% of the cluster concentration, consistent with the vast majority of the cluster being EPR silent, *i.e.* as [4Fe–4S]^2+^. Addition of sodium dithionite led to virtually complete loss of the [3Fe–4S] signal, consistent with reduction to the EPR-silent state, [3Fe–4S]^0^. No evidence for a reduced [4Fe–4S]^1+^ form was observed. Gel filtration of reconstituted RirA under anaerobic conditions resulted in an increase in the [3Fe–4S]^1+^ signal in the EPR spectrum to ∼13% of the total cluster concentration. Since the [3Fe–4S] form arises from loss of iron from the [4Fe–4S] cluster, this suggested that the cluster is not particularly stable under the conditions of gel filtration in which dissociated iron was separated from the protein-bound cluster. Addition of O_2_ (by exposure to air, 230 μM final concentration) or potassium ferricyanide (100 μM final concentration) led to increases in the *g* = 2.01 EPR signal (due to a [3Fe–4S]^1+^ form) after 30 min to ∼11% and ∼39% of the original cluster concentration, respectively (Fig. 3C). The effects of a range of O_2_ concentrations (58–230 μM) were examined by monitoring cluster degradation *via* absorbance at 383 nm (Fig. 3D). These traces revealed that the rate of reaction with O_2_ increased along with O_2_ concentration. Each of the traces could be fitted with a single exponential function, which revealed a rate constant, *k* = 0.019 ± 0.004 min^–1^ (Table 2), that was essentially independent of the O_2_ concentration, consistent with the rate-limiting step of the reaction not involving O_2_. Thus, the slow step of cluster disassembly is not the reaction with O_2_.

**Table 2:** Rate constants for O_2_ and chelator-promoted [4Fe–4S] cluster conversion/degradation at 25 °C

Iron chelator	Aerobic/anaerobic	Rate constant (min^–1^)
—	+O_2_ (59–230 μM)	1.9 (±0.4) × 10^–2^

**EDTA**		
1 mM	–O_2_	2.6 (±0.4) × 10^–3^
	+O_2_ (230 μM)	8.5 (±0.4) × 10^–3^
4 mM	–O_2_	5.0 (±0.8) × 10^–3^
Chelex	–O_2_	2.4 (±0.4) × 10^–3^
	+O_2_ (230 μM)	6.1 (±0.7) × 10^–2^

### Low iron conditions promotes [4Fe–4S] to [2Fe–2S] cluster conversion and subsequent cluster loss

**Fig. 4:**
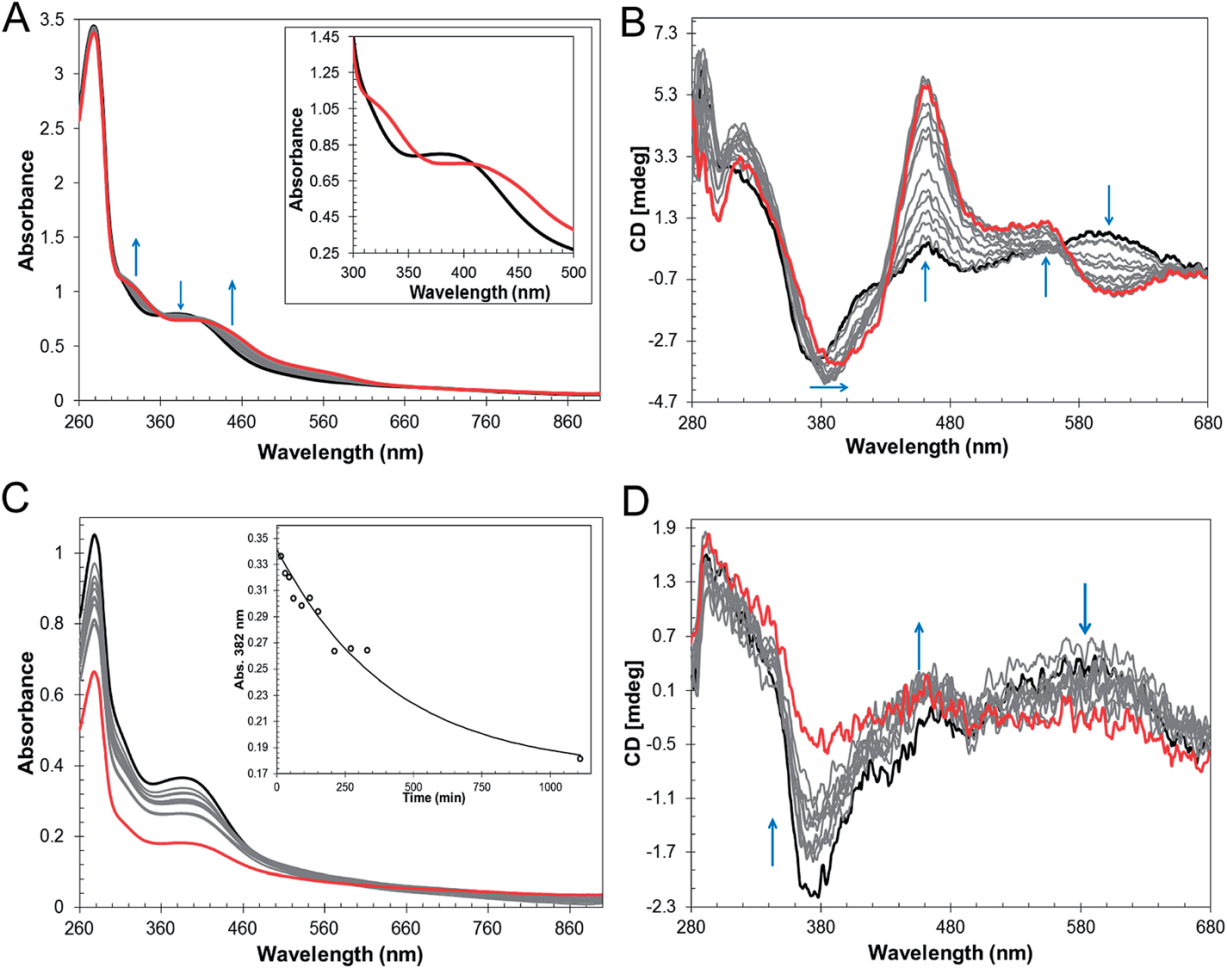
Response of [4Fe–4S] RirA to low iron conditions. (A) UV-visible absorbance spectra and (B) CD spectra of [4Fe–4S] RirA (77 μM in cluster in buffer B) following addition of increasing concentrations of EDTA (up to 8.2 mM) under anaerobic conditions. Starting and end-point spectra are in black and red, respectively. Inset in (A) are absorbance spectra in the absence of EDTA (black) and with 8.2 mM EDTA (red). (C) and (D) as in (A) and (B), except that Chelex-100, separated from the protein by dialysis membrane, was the iron chelator. Inset in (C) is a plot of *A*_382 nm_ as a function of time; the solid line represents a fit of the data (*k* = 2.4 ± 0.6 × 10^–3^ min^–1^). In (B) and (D), arrows show the most significant changes in spectral features during the titration. Pathlength was 1 cm for all measurements.

The mechanism by which RirA senses iron and therefore how regulation is achieved is unknown but, *a priori*, seems likely to involve its iron–sulfur cluster. As gel filtration resulted in a significant increase in the [3Fe–4S]^1+^ component, this suggested that the [4Fe–4S] cluster of RirA is susceptible to loss of iron if there is a means to separate it from the residual cluster. To further probe the response of the cluster to low iron conditions, [4Fe–4S] RirA was exposed to the iron chelator EDTA under anaerobic conditions. A titration of RirA with increasing concentrations of EDTA was followed using UV-visible and CD spectroscopies. The absorbance changes (Fig. 4A) indicate the conversion of the [4Fe–4S] cluster, with a red shift of the 383 nm band and significant absorbance emerging in the 500–600 nm region characteristic of a [2Fe–2S] cluster.^18^,^20^ The CD changes (Fig. 4B) also clearly reflected the cluster conversion process, with a red shift of the (–)382 nm band, and the emergence of new bands at (+)464 nm, (+)540 nm and (–)585 nm. The CD spectrum of RirA following exposure to EDTA is similar to those of characterised [2Fe–2S] cluster proteins, for example the H64D variant of the Rieske-type [2Fe–2S] ferredoxin from *Sulfolobus solfataricus*,^40^ and the monothiol glutaredoxins GrxS16 from *Arabidopsis thaliana*, and GrxS14 and GrxC1 from *Populus trichocarpa*.^41^ A very similar cluster conversion process was observed by CD when Ferrozine was used as the chelator (Fig. S6†), indicating that the observed effect is not chelator-specific.

**Fig. 5:**
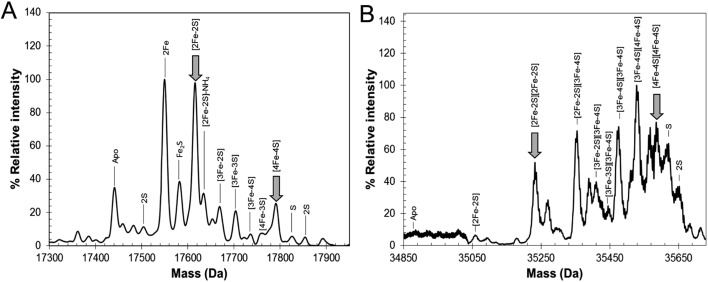
ESI-MS analysis of RirA following low iron-mediated cluster conversion. Positive ion mode ESI-TOF native mass spectra of ∼21 μM [4Fe–4S] RirA in 250 mM ammonium acetate pH 7.35 following treatment with 1 mM EDTA for 2.5 h. Deconvoluted spectrum in the (A) monomer and (B) dimer mass regions. ESI-MS data for RirA prior to the addition of EDTA are shown in Fig. 3.

To further establish the nature of the cluster conversion process that RirA undergoes under low iron conditions, ESI-MS under non-denaturing conditions was again employed. Treatment of [4Fe–4S] RirA with EDTA and subsequent removal of the chelator generated the same converted form of RirA as that described above. The *m*/*z* spectrum again revealed the presence of monomeric and dimeric forms of RirA (Fig. S7†). The deconvoluted mass spectrum in the monomer region (Fig. 5A) featured two main peaks, corresponding to RirA containing a [2Fe–2S] cluster and to RirA containing two irons (see Table 1), which represents a breakdown product of the [2Fe–2S] cluster. As expected from the earlier spectrophotometric observations, the peak due to [4Fe–4S] RirA was of much lower intensity. Other [4Fe–4S] cluster breakdown species, corresponding to [3Fe–4S], [3Fe–3S] and [3Fe–2S] clusters, were also observed, along with [2Fe–S] and apo-RirA (Fig. 5A), but all of these were at low abundance relative to the [2Fe–2S] form.

In the dimer region, the signal to noise was relatively poor but peaks were still clearly present (Fig. 5B), including those due to the RirA dimer containing two [2Fe–2S] clusters and two [4Fe–4S] clusters (Table 1). Various cluster breakdown forms were also present, including [3Fe–4S]/[4Fe–4S], [3Fe–4S]/[3Fe–4S], [3Fe–3S]/[3Fe–3S] and [2Fe–3S]/[3Fe–3S] RirA, consistent with conversion of [4Fe–4S] into [2Fe–2S] clusters. Again, the dimer region contained fewer breakdown products (between the [2Fe–2S] and apo-RirA species). For example, there was no evidence of RirA containing two irons (observed in the monomer region), consistent with the suggestion that the cluster is more stable within the dimer form of RirA during the MS experiment.

The rate of cluster conversion under anaerobic conditions upon addition of 1 or 4 mM EDTA was investigated by UV-visible absorbance and CD spectroscopies, Fig. S8.† The same changes that occurred in the thermodynamic titration experiments were observed, indicating cluster conversion. However, these occurred relatively slowly. Absorbance data at 382 nm were fitted with a single exponential (Fig. S8†), giving rate constants of ∼0.003 min^–1^ and ∼0.005 min^–1^, respectively, for 1 and 4 mM EDTA (Table 2). Removal of EDTA by passage of the sample down a gel filtration column did not affect the shape of the absorbance or CD spectra, consistent with a stable [2Fe–2S] product of cluster conversion (*e.g.* Fig. S8A†). Prolonged (overnight) exposure of [2Fe–2S] RirA to EDTA led to significant loss of the cluster such that apo-RirA was the principal form of the protein (*e.g.* Fig. S8†).

In the above experiments, EDTA and Ferrozine were in the same solution as RirA and therefore could potentially interact directly with RirA to promote cluster conversion in a non-physiological reaction. To investigate cluster conversion in the absence of direct interaction with a chelator, experiments were performed in which a solid chelating resin (Chelex 100) was separated from the protein by a semi-permeable membrane. Under anaerobic conditions, absorbance (Fig. 4C) and CD (Fig. 4D) intensity due to the cluster was lost gradually over several hours, but with formation of only small amounts of [2Fe–2S], showing that under these conditions apo-RirA was formed without stabilization of [2Fe–2S] RirA. Absorbance decay at 382 nm was fitted with a single exponential, giving a rate constant of ∼0.002 min^–1^ (Fig. 4C and Table 2).

Thus, in all cases, in the presence of a chelator, the RirA cluster was found to be not only susceptible to the loss of iron, but also to undergo a cluster conversion process to form a transient [2Fe–2S] species that itself was unstable (under low iron conditions), leading to further breakdown to apo-RirA. For reasons that are not clear, the extent of the stability of the [2Fe–2S] form varied according to the particular chelator. Thus, in the presence of EDTA and Ferrozine, it was readily observed and could be stabilized under anaerobic conditions following removal of the chelator. However, with Chelex 100, it was less stable and did not accumulate as a distinct intermediate. Although we do not have information on the cluster configuration of RirA in *R. leguminosarum* itself, anaerobically purified RirA generated from heterologous expression in aerobically grown *E. coli* contained a mixture of both [2Fe–2S] and [4Fe–4S] clusters (Fig. S1†). This supports a mechanism in which cluster transformation occurs in the cell.

The sensitivity of the [4Fe–4S] cluster of RirA to low iron conditions raised the question of whether this is a general property of Rrf2 family regulators that bind a [4Fe–4S] cluster. To gain some insight into this, equivalent experiments using Chelex 100 were performed with the [4Fe–4S] cluster form of dimeric NsrR, another Rrf2 family regulator. In contrast to RirA, no significant cluster loss was observed for NsrR over a 2 h period (Fig. S9†). This suggests that cluster fragility/conversion is a physiologically important characteristic of [4Fe–4S] RirA and is not a general trait of Rrf2 regulators.

### Enhanced rate of cluster conversion/loss under aerobic low iron conditions

Given the sensitivity of [4Fe–4S] RirA to separate exposure to low iron and O_2_, the combined effects of low iron and saturating levels of O_2_ on [4Fe–4S] RirA were investigated. Addition of 1 mM EDTA to [4Fe–4S] RirA under aerobic conditions (230 μM O_2_) resulted in a similar reaction to that under anaerobic conditions, but cluster breakdown occurred more rapidly. Both absorbance and CD (Fig. S10A and B†) revealed the transient formation of the [2Fe–2S] form, which rapidly decayed further to form apo-RirA as the final product. Fitting of absorbance data at 382 nm gave a rate constant of ∼0.01 min^–1^, ∼3-fold higher than under anaerobic conditions. Under the low iron conditions generated by Chelex 100 resin, the presence of O_2_ (230 μM) also increased the rate of reaction, with a rate constant (∼0.06 min^–1^) ∼30-fold higher than under anaerobic conditions (Table 2). Both absorbance and CD data (Fig. S10C and D†) were consistent with the transient formation of a [2Fe–2S] form before decay to the apo-protein. Thus, iron insufficiency and the presence of O_2_ results in an enhanced rate of cluster conversion/degradation, and the interplay between these conditions controls the rate of cluster reaction.

### O_2_ affects expression of RirA-regulated genes in *R. leguminosarum*

**Fig. 6:**
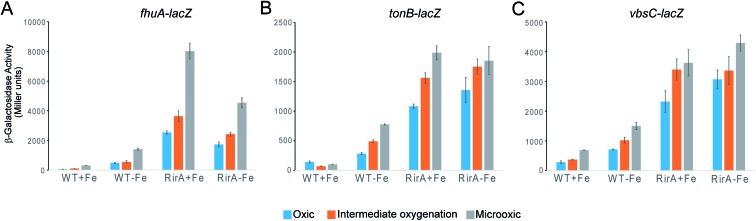
Expression analysis of the effect of iron and O_2_ on RirA-regulated genes. β-Galactosidase assays with wild type (WT) and *rirA* mutant (RirA) strains of *R. leguminosarum* containing *fhuA*, *tonB lacZ* or *vbsC* fusion plasmids, grown in high-Fe (+Fe) or low-Fe (–Fe) minimal medium under oxic, intermediate oxygenation and microoxic conditions. Activities are shown in Miller units with standard errors indicated by error bars (*n* = 3).

The *in vitro* sensitivity of RirA [4Fe–4S] to O_2_ suggested that the regulatory function of RirA should be affected by O_2_ levels in *R. leguminosarum* cultures, as well as by the previously documented availability of iron.^6^–^9^ To investigate the effect of O_2_ on RirA-regulated gene expression *in vivo*, β-galactosidase assays were performed using *fhuA-lacZ*, *tonB-lacZ* and *vbsC-lacZ* fusion constructs^42^ in wild type *R. leguminosarum* and in a *rirA* deficient mutant, each grown with high or low iron availability under aerobic and microoxic conditions, and at intermediate oxygenation, see Fig. 6. As expected, expression levels of *fhuA*, *tonB*, and *vbsC* were significantly upregulated under low iron in wild type *R. leguminosarum* (as previously reported^6^), but in the *rirA* mutant their expression was at high level in both iron regimes. We also note the elevated expression in the *rirA* mutant compared to wild type under low iron (as previously reported),^6^ indicating that RirA remains active as a repressor even when iron levels are low. However, the data also revealed the previously unrecognised RirA-independent regulation of *fhuA*, *tonB* and *vbsC* expression in response to O_2_; thus, in both the wild type and *rirA* mutant, expression levels were reduced as the O_2_ concentration increased. This indicates that the cell minimises further iron uptake when O_2_ levels are high, consistent with the need to counter Fenton-mediated toxicity of iron plus O_2_. This additional O_2_-dependent, but RirA-independent, regulation likely masks any direct effect of O_2_ on RirA and so conclusions about the effect of O_2_ on RirA *in vivo* cannot be drawn. While the mechanism by which O_2_ exerts its regulatory effect is unknown, these data are important because they demonstrate that iron and O_2_ regulation are intertwined in *R. leguminosarum* and indicate that iron regulation is highly complex, involving a combination of regulatory mechanisms. We note that the expression of the RirA-regulated *sufS2* gene of *Agrobacterium tumefaciens* was derepressed under conditions of oxidative stress,^15^ suggesting that O_2_/ROS sensitivity is a general feature of RirA. Furthermore, Irr-mediated regulation is also sensitive to oxidative stress,^28^ suggesting that this could be a general feature of iron regulation.

### [4Fe–4S] RirA, but not apo-RirA, binds RirA-regulated *fhuA* IRO box operator DNA

**Fig. 7:**
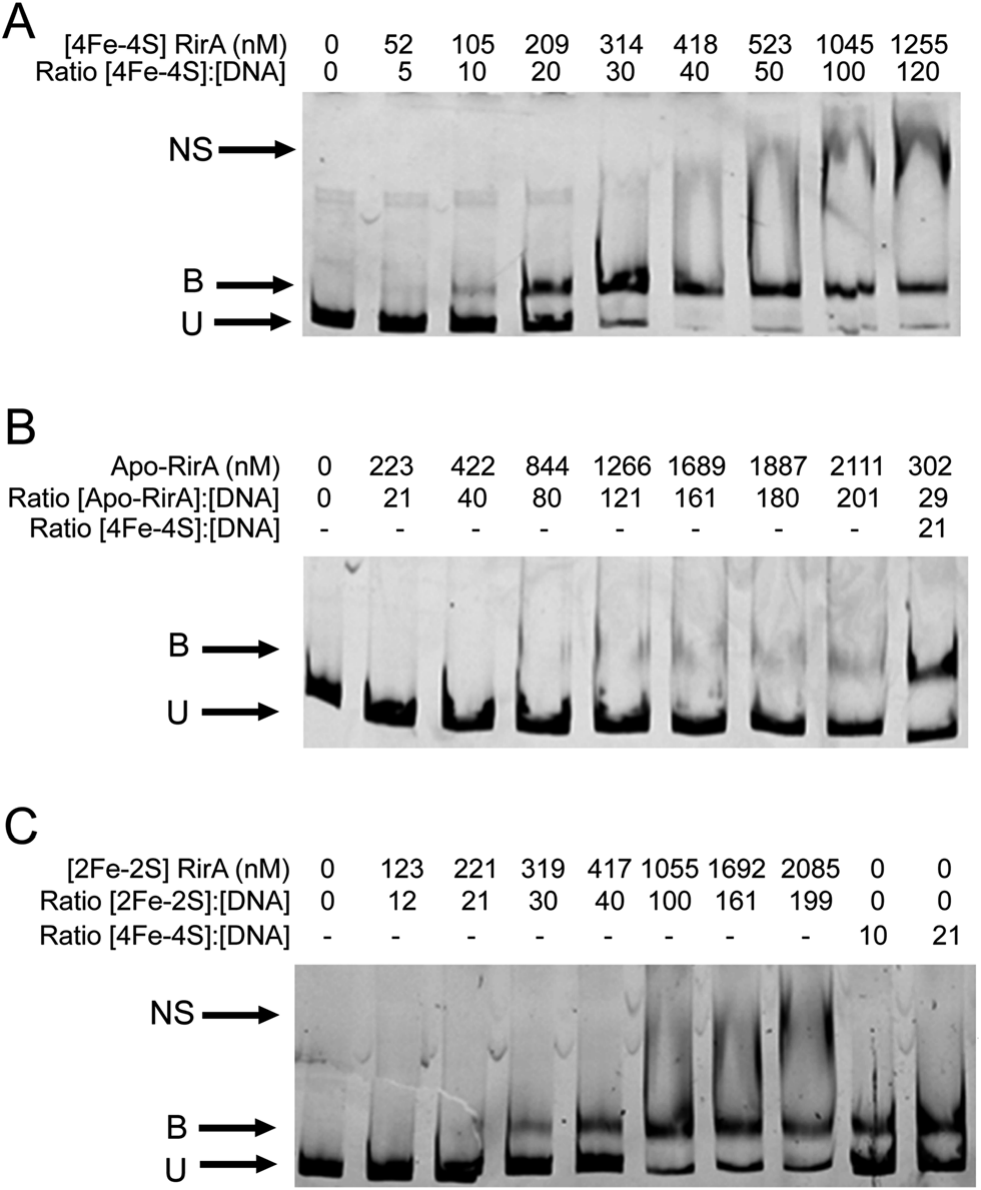
Cluster-dependent DNA binding by RirA. EMSAs showing the *fhuA* promoter DNA probe in unbound (U), bound (B), and non-specifically bound (NS) forms by (A) [4Fe–4S] RirA, (B) apo-RirA and (C) [2Fe–2S] RirA. Ratios of [4Fe–4S]/[2Fe–2S] RirA and [RirA] to DNA are indicated. The binding buffer contained 10 mM Tris, 60 mM KCl, pH 7.52.

EMSAs were carried out with [4Fe–4S] RirA using a fragment (see Fig. S11†) that spanned the IRO box operator, plus the *fhuA* promoter, which is known to be under the control of RirA *in vivo*,^6^,^9^ see Fig. 7. A titration with increasing concentrations of [4Fe–4S] RirA resulted in increased levels of bound DNA, with essentially full binding observed at a [4Fe–4S] RirA : DNA ratio of 40 : 1. At higher protein levels, non-specific binding was observed (Fig. 7A). Analysis of binding using densitometry provided an estimate of the *K*_d_ for the [4Fe–4S] RirA–DNA complex of ∼170 nM (Fig. S12† and Table 3). Importantly, an equivalent experiment with apo-RirA (Fig. 7B and S12†) revealed much weaker DNA-binding, with *K*_d_ > 5 μM (*i.e.* >30-fold lower affinity than the [4Fe–4S] form), demonstrating that the [4Fe–4S] form is the active form for DNA-binding.

**Table 3:** Dissociation constants (*K*_d_) for RirA–DNA complex formation

RirA	*K*_d_ for binding to *fhuA* promoter region (nM)
[4Fe–4S]	172 (±57)
[2Fe–2S]	482 (±97)
Apo	>5500

Observations of a [2Fe–2S] form of RirA (above) raised the question of whether such a form can bind DNA. EMSA experiments with the *fhuA* promoter were repeated using [2Fe–2S] RirA, Fig. 7C. Although non-specific binding was observed at higher ratios of protein to DNA, the data clearly show that the [2Fe–2S] form binds DNA significantly more weakly than the [4Fe–4S] form. Analysis by densitometry gave an estimate of the *K*_d_ for the [2Fe–2S] RirA–DNA complex of 482 ± 97 nM (Fig. S12†), suggesting that the [4Fe–4S] form binds ∼3-fold more tightly. We also note the possibility that a residual amount of [4Fe–4S] cluster form could contribute to the observed binding.

### Comparison between RirA and other iron–sulfur cluster regulatory proteins

The behavior of RirA draws comparison with other iron–sulfur cluster-containing regulators. For example, FNR is an O_2_ sensor that controls the switch between anaerobic and aerobic metabolism. This is mediated through reaction of its [4Fe–4S] cluster with O_2_, which promotes conversion to a [2Fe–2S] cluster form that can no longer bind DNA.^29^,^36^,^43^ Despite the lack of any sequence or structural similarities between FNR and RirA, there are clear similarities in terms of the cluster conversion reactions (in the case of RirA, mediated by the synergistic influences of iron and O_2_).

The properties of RirA described here are also clearly related to those of the Rrf2 superfamily member IscR,^44^ which in *E. coli* controls the expression of approx. 40 genes, including the *isc* and *suf* iron–sulfur cluster biosynthesis operons.^45^,^46^ Unusually, IscR binds to two types of promoters (type 1 and 2); binding to type 1 is dependent on the presence of a [2Fe–2S] cluster, whereas binding to type 2 promoters is independent of the cluster (apo-IscR binds as tightly as [2Fe–2S] IscR).^45^,^46^ Under conditions where there is sufficient iron–sulfur cluster supply, [2Fe–2S] IscR binds type 1 promoters and represses Isc iron–sulfur cluster biogenesis. When iron–sulfur cluster supply is insufficient, apo-IscR is formed and cluster biosynthesis is de-repressed. Iron–sulfur cluster demand varies and is higher under aerobic than anaerobic conditions, particularly under oxidative stress, where turnover of iron–sulfur clusters in the cell is higher. Under these conditions, apo-IscR is the predominant form and can bind type 2 promoters to inhibit expression of anaerobic iron–sulfur cluster containing respiratory proteins and activate Suf iron–sulfur cluster biosynthesis. Thus, there is a complex interplay between iron–sulfur cluster demand and turnover due to O_2_/oxidative stress.^17^,^45^–^47^ We note that the Rrf2 family NO-responsive regulator NsrR from *Bacillus subtilis* has also been shown to recognize two types of promoter sites, only one of which is cluster-dependent.^48^

**Fig. 8:**
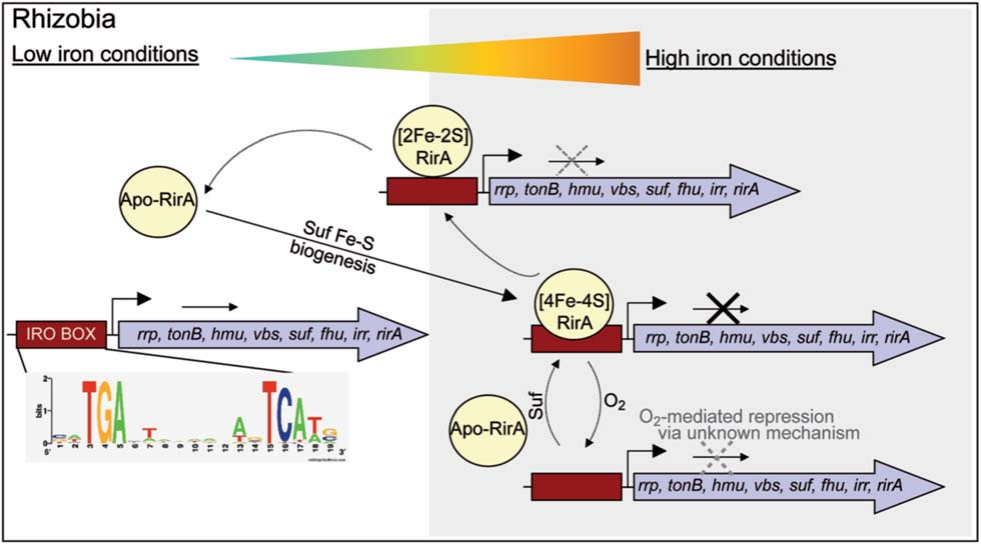
RirA mediated regulation of iron-responsive genes in *Rhizobium*. Under iron sufficient conditions, RirA accommodates a [4Fe–4S] cluster (*via* the Suf system) and binds to the IRO motif (sequence shown) present in the promoter regions of RirA-regulated genes, repressing their transcription. In low iron medium, the [4Fe–4S] cluster of RirA is unstable to conversion/degradation, yielding apo-RirA *via* a [2Fe–2S] form. Apo-RirA does not bind the IRO motif and so these genes are no longer repressed by RirA. [2Fe–2S] RirA retains some ability to (weakly) bind the IRO motif, resulting in a partial alleviation of repression under conditions of mild iron deficiency. O_2_/oxidative stress destabilizes the RirA cluster, leading to increased turnover of [4Fe–4S] RirA even under iron-replete conditions. We note that specific, as yet unidentified, cellular factors might also be involved in, and influence the rate of, cluster degradation.

While there is no evidence for more than one type of DNA-binding site, our data on RirA indicate some similarities to IscR. The significant sensitivity of RirA to O_2_ suggests that even when iron is sufficient, the protein is susceptible to cluster conversion/loss.^47^ We note that the Suf iron–sulfur cluster biosynthetic machinery of *R. leguminosarum* is under RirA regulation (and is also regulated by Irr).^9^,^10^ Under iron sufficiency, iron–sulfur cluster biosynthesis is still required for multiple processes in the cell. The continuous breakdown of [4Fe–4S] RirA mediated by O_2_ is likely to be important for the cell to maintain iron–sulfur cluster biosynthesis. Because iron–sulfur cluster biosynthesis is also required for RirA-mediated repression, this provides a mechanism to ensure the cellular demand for iron–sulfur clusters is met. The data are consistent with RirA functioning as a sensor of iron *via* iron–sulfur cluster availability (see Fig. 8), rather than as a direct sensor of Fe^2+^ through, for example, a [4Fe–4S]^2+^ ↔ [3Fe–4S]^1+^ equilibrium dependent on Fe^2+^ ion availability, or through reversible switching between [4Fe–4S] and [2Fe–2S] forms, though this possibility cannot be ruled out.

## Conclusions

RirA is a novel type of iron sensor that is wholly distinct from the well-characterized Fur and DtxR proteins, which sense Fe^2+^ directly,^2^,^4^ and from Irr, another Fur family regulator, which senses heme.^27^ Here we have shown that reconstitution of RirA generates a [4Fe–4S] cluster form, and that this binds DNA containing an IRO motif but apo-RirA does not. Under low iron conditions, the RirA cluster was found to be not only susceptible to the loss of iron, but also to undergo a cluster conversion process to form a transient [2Fe–2S] species that itself was unstable (under low iron conditions), leading to further breakdown to apo-RirA. Though some binding of [2Fe–2S] RirA to IRO motif DNA was observed, this was ∼3-fold weaker than that of [4Fe–4S] RirA. These observations raise the possibility that the [2Fe–2S] form of RirA is part of a sliding scale response to iron availability: under iron sufficiency, the [4Fe–4S] form tightly binds IRO motifs at IRO sequences and represses the RirA regulon. Under moderately low iron, [2Fe–2S] RirA binds weakly to IRO motifs, partially repressing the regulon; and under iron insufficiency, apo-RirA cannot bind the IRO motif and the regulon is entirely de-repressed (Fig. 8). The enhanced sensitivity of [4Fe–4S] RirA to low iron under aerobic conditions and the observed effect of O_2_ levels on expression of RirA-regulated genes in *R. leguminosarum* cultures is consistent with a complex interplay between iron–sulfur cluster demand and turnover due to O_2_/oxidative stress, and the need to minimize iron uptake under O_2_/oxidative stress conditions.^17^,^45^–^47^ Overall, our observations indicate that [4Fe–4S] RirA is the principal form of the protein under iron-replete conditions, accounting for how it functions as a repressor.^6^,^9^

Our work on RirA and Irr,^27^,^49^ the two global iron regulators of Rhizobia, point to a mechanism of iron-responsive gene regulation that is more subtle and integrative than is believed to occur in bacteria that sense iron directly *via* Fe^2+^.^50^ By binding an iron–sulfur cluster and heme, respectively, RirA and Irr sense iron levels indirectly by monitoring two physiological important iron-containing molecular signals, whose intracellular concentrations respond to external iron concentrations. Importantly, iron–sulfur cluster and heme levels likely vary according to other criteria, such as growth rate, O_2_ levels, carbon source *etc.*, so the management of iron resources in the cell may be matched to its overall physiological status. Although highly conserved versions of RirA are only found in a taxonomically restricted group of alpha Proteobacteria, these include several pathogens of animals (*Brucella*, *Bartonella*) and plants (*Agrobacterium*). Thus, these new findings on this novel regulator will likely find applicability to these, and other important bacterial genera.

## Materials and methods

### Preparation of [4Fe–4S], [2Fe–2S] and apo-RirA

Luria-Bertani medium (8 × 625 mL) was inoculated with freshly transformed BL21 λDE3 *E. coli* containing the expression vector pET11a with the *rirA* gene (CAC35510) from *R. leguminosarum* strain 8401 (pRL1JI) obtained from GenScript with optimization for *E. coli* codon usage. Ampicillin (100 μg mL^–1^) and ferric ammonium citrate (20 μM) were added and the cultures grown at 37 °C, 200 rpm until OD_600 nm_ was 0.6–0.9.^51^ To facilitate *in vivo* iron–sulfur cluster formation,^51^ the flasks were placed on ice for 18 min, then expression was induced with 7.5 μM IPTG with incubation at 30 °C and 105 rpm. After 50 min, the cultures were supplemented with 200 μM ammonium ferric citrate and 25 μM l-methionine (to provide additional sulfur) and incubated for a further 3.5 h at 30 °C. The cells were harvested by centrifugation at 10 000 × *g* for 15 min at 4 °C. Unless otherwise stated, all subsequent purification steps were performed under anaerobic conditions inside an anaerobic cabinet (O_2_ < 2 ppm). Cell pellets were resuspended in 70 mL of buffer A (25 mM HEPES, 2.5 mM CaCl_2_, 50 mM NaCl, pH 7.5) to which were then added 30 μg mL^–1^ of lysozyme and 30 μg mL^–1^ of phenylmethane sulfonyl fluoride. The cell suspension was thoroughly homogenized by syringe, removed from the anaerobic cabinet, sonicated twice while on ice, and returned to the anaerobic cabinet. The cell suspension was transferred to O-ring sealed centrifuge tubes (Nalgene) and centrifuged outside of the cabinet at 40 000 × *g* for 45 min at 1 °C.

The supernatant was passed through a HiTrap Heparin (1 × 5 mL; GE Healthcare) column using an ÄKTA Prime system at 1 mL min^–1^. The column was washed with Buffer A until *A*_280 nm_ < 0.1. Bound proteins were eluted using a 100 mL linear gradient from 0 to 100% buffer B (25 mM HEPES, 2.5 mM CaCl_2_, 50 mM NaCl, 750 mM KCl, pH 7.5). Fractions containing RirA were pooled and stored in an anaerobic freezer until needed. *In vitro* cluster reconstitution to generate [4Fe–4S] RirA was carried out in the presence of NifS, as described previously.^52^ For cluster lability control experiments, *Streptomyces coelicolor* [4Fe–4S] NsrR was purified and assayed for cluster content as previously described.^20^ To prepare [2Fe–2S] RirA, [4Fe–4S] RirA was diluted with buffer B and incubated with 1 mM ethylenediaminetetraacetate (EDTA) for 2.5 h. A desalting column (PD10, GE Healthcare) was used to remove the EDTA. Under these conditions, [2Fe–2S] RirA was stable for several weeks at 4 °C. Apo-RirA was prepared from as isolated holoprotein by aerobic incubation with 1 mM EDTA overnight.

### O_2_ and iron chelator experiments

To determine the sensitivity of [4Fe–4S] RirA to O_2_, the protein was placed in an anaerobic cuvette and rapidly diluted with aerobic buffer B to give the desired O_2_ concentration and the cluster response was followed by spectroscopy. To simulate low iron conditions, the soluble iron chelators EDTA or Ferrozine were added at variable concentration to solutions of [4Fe–4S] RirA in buffer B and the cluster response followed *via* spectroscopy. The affinities of these chelators for iron are high (Fe^2+^–EDTA), log *K* = 14.3, Fe^3+^–EDTA, log *K* = 25.1;^53^ Fe^2+^(Ferrozine)_3_, log *K* (β_3_) = 15.4 (ref. 54) and under the experimental conditions used here, free iron concentrations were limited to sub-femtomolar levels, thus providing efficient competition for cluster-derived iron. For titrations, solutions were incubated for 15 min between additions. For kinetic experiments, reactions were started by rapid addition and mixing of the chelator and protein to give the desired final concentrations at 25 °C. For experiments with the insoluble metal chelator Chelex 100, which contains the immobilized iminodiacetic acid (IDA) group (Fe^2+^–IDA, log *K* = 5.8; Fe^3+^–IDA, log *K* = 10.9),^55^ the resin (20 g) was placed in the bottom of a flask containing buffer B (125 mL) and a dialysis cassette (5 mL, Spectra/Por® Float-A-Lyzer G2 Biotech) containing 30 μM of [4Fe–4S] RirA was placed in the solution. Cluster response was monitored by periodically transferring the protein solution to a cuvette for spectrophotometric measurement. For similar experiments with [4Fe–4S] NsrR from *Streptomyces coelicolor*, the protein was purified as previously described.^20^

### Spectroscopy

UV-visible absorbance measurements were performed using a Jasco V500 spectrometer, and CD spectra were measured with a Jasco J810 spectropolarimeter. EPR measurements were made with an X-band Bruker EMX EPR spectrometer equipped with a helium flow cryostat (Oxford Instruments). EPR spectra were measured at 10 K at the following instrumental settings: microwave frequency, 9.471 GHz; microwave power, 3.18 mW; modulation frequency, 100 kHz; modulation amplitude, 5 G; time constant, 82 ms; scan rate, 22.6 G s^–1^; single scan per spectrum. Relative concentrations of the paramagnetic species were measured using the procedure of spectral subtraction with a variable coefficient^56^ and converted to absolute concentrations by comparing an EPR spectrum second integral to that of a 1 mM Cu(ii) in 10 mM EDTA standard, at non-saturating values of the microwave power.

### Mass spectrometry

For mass spectrometry under non-denaturing conditions, [4Fe–4S] RirA was exchanged into 250 mM ammonium acetate, pH 7.3, using a desalting column (PD-10, GE Healthcare), diluted to ∼30 μM cluster and infused directly (0.3 mL h^–1^) into the ESI source of a Bruker micrOTOF-QIII mass spectrometer (Bruker Daltonics, Coventry, UK) operating in the positive ion mode, and calibrated using ESI-L Low Concentration Tuning Mix (Agilent Technologies, San Diego, CA). Mass spectra (*m*/*z* 500–1750 for RirA monomer; *m*/*z* 1800–3500 for RirA dimer) were acquired for 5 min using Bruker oTOF Control software, with parameters as follows: dry gas flow 4 L min^–1^, nebuliser gas pressure 0.8 bar, dry gas 180 °C, capillary voltage 2750 V, offset 500 V, ion energy 5 eV, collision RF 180 Vpp, collision cell energy 10 eV. Optimization of experimental conditions for the transmission of dimeric species was achieved by increasing the capillary voltage to 4000 V and the collision RF to 600 Vpp.^57^

Processing and analysis of MS experimental data were carried out using Compass DataAnalysis version 4.1 (Bruker Daltonik, Bremen, Germany). Neutral mass spectra were generated using the ESI Compass version 1.3 Maximum Entropy deconvolution algorithm over a mass range of 17 300–18 000 Da for the monomer and 34 850–35 810 Da for the dimer. Exact masses are reported from peak centroids representing the isotope average neutral mass. For apo-proteins, these are derived from *m*/*z* spectra, for which peaks correspond to [*M* + *nH*]^*n*+^/*n*. For cluster-containing proteins, where the cluster contributes charge, peaks correspond to [*M* + (Fe–S)^*x*+^ + (*n* – *x*)*H*]^*n*+^/*n*, where *M* is the molecular mass of the protein, Fe–S is the mass of the particular iron–sulfur cluster of *x*+ charge, *H* is the mass of the proton and *n* is the total charge. In the expression, the *x*+ charge of the iron–sulfur cluster offsets the number of protons required to achieve the observed charge state (*n*+).^35^ Predicted masses are given as the isotope average of the neutral protein or protein complex, in which iron–sulfur cluster-binding is expected to be charge-compensated.^33^,^58^

### Electrophoretic mobility shift assays (EMSAs)

A DNA fragment (581 bp, see Fig. S10†) carrying the *fhuA* promoter was PCR-amplified using genomic DNA with 5′ 6-carboxyfluorescein (FAM)-modified primers (Eurofins). The PCR products were extracted and purified using a QIAquick gel extraction kit (Qiagen) according to the manufacturer's instructions. Probes were quantitated using a nanodrop ND2000c. The molecular weights of the double-stranded FAM-labelled probes were calculated using OligoCalc.^43^ Bandshift reactions (20 μL) were carried out on ice in 10 mM Tris, 60 mM KCl, pH 7.52. Briefly, 1 μL of DNA was titrated with varying aliquots of RirA. Loading dye (2 μL, containing 0.1% {w/v} bromophenol blue) was added and the reaction mixtures were immediately separated at 30 mA on a 7.5% (w/v) polyacrylamide gel in 1× TBE (89 mM Tris,89 mM boric acid, 2 mM EDTA), using a Mini Protean III system (Bio-Rad). Polyacrylamide gels were pre-run at 30 mA for 2 min prior to use. Gels were visualized (excitation, 488 nm; emission, 530 nm) on a molecular imager FX Pro (Bio-Rad). Densitometric analysis was performed using Image Studio Lite (Li-Cor Biotechnology) and the resulting data fitted using a simple binding equation in Origin 8 (Origin Labs).

### Other analytical techniques

Protein concentrations were determined using the method of Bradford (Bio-Rad), with bovine serum albumin as the standard. Cluster concentrations were determined by iron and sulfide assays,^59^,^60^ from which an absorbance extinction coefficient at 383 nm for the RirA [4Fe–4S] cluster was determined as 13 460 ± 250 M^–1^ cm^–1^. Kinetic data at *A*_386 nm_ were recorded *via* a fibre optic link, as previously described.^29^ Gel filtration was carried out under anaerobic conditions using a Sephacryl S-100HR 16/50 column (GE Healthcare), equilibrated in buffer B with a flow rate of 1 mL min^–1^.

### β-galactosidase assays

*E. coli* was grown in Luria-Bertani (LB)^61^ complete medium at 37 °C. *R. leguminosarum* was grown in tryptone yeast (TY)^62^ complete medium, high-Fe Y^62^ minimal medium with 10 mM succinate as carbon source and 10 mM NH_4_Cl as nitrogen source, or low-Fe Y minimal medium with 10 mM succinate as carbon source and 10 mM NH_4_Cl as nitrogen source and 20 μM 2,2′-dipyridyl, at 28 °C and 150 rpm shaking (oxic conditions), 75 rpm shaking (intermediate oxygenation) or no shaking (microoxic conditions). Where necessary, antibiotics were added to media at the following concentrations: streptomycin (400 μg mL^–1^) and tetracycline (200 μg mL^–1^).

The plasmids pBIO1125 (Tc^r^), pBIO1247 (Tc^r^) and pBIO1306 (Tc^r^), which contain the *E. coli lacZ* reporter gene under the control of the *R. leguminosarum fhuA*,^6^*tonB*,^63^ and *vbsC*,^64^ promoters, respectively, were conjugated from *E. coli* to *R. leguminosarum* by triparental mating using helper plasmid pRK2013.^65^ Starting cultures of *R. leguminosarum* with or without pBIO1125, pBIO1247 and pBIO1306 were grown in TY complete medium until OD_600_ was 0.6, then 1 mL was washed and transferred to 100 mL of either high-Fe Y minimal medium low-Fe Y minimal medium and incubated under oxic, intermediate oxygenation or microoxic conditions. After 24 hours, 50 mL of the intermediate oxygenation cultures were transferred to Falcon 50 mL conical centrifuge tubes and incubated at 28 °C and microoxic conditions. β-Galactosidase assays were performed after another 24 hours for both high and low Fe cultures under oxic, intermediate oxygenation and microoxic conditions, as described by Miller.^66^

## Conflicts of interest

There are no conflicts of interest to declare.

## Abbreviations

CD: Circular dichroism
EDTA: Ethylenediaminetetraacetate
ESI-MS: Electrospray ionization mass spectrometry (ESI-MS)
EMSA: Electrophoretic mobility shift assay
EPR: Electron paramagnetic resonance
IPTG: Isopropyl β-d-1-thiogalactopyranoside
HEPES: 4-(2-Hydroxyethyl)piperazine-1-ethanesulfonic acid
UV: Ultraviolet

## Supplementary Material

Supplementary information
