# Supplementary material for: Sensing iron availability via the fragile [4Fe–4S] cluster of the bacterial transcriptional repressor RirA
Source: Chem Sci. 2017 Oct 23;8(12):8451–63. doi: 10.1039/c7sc02801f (PMC5863699; doi:10.1039/c7sc02801f)
Supplement: Supplementary file 1 [file SC-008-C7SC02801F-s001.pdf]

Electronic Supplementary Material (ESI) for Chemical Science.
This journal is © The Royal Society of Chemistry 2017

Electronic Supplementary Information

Sensing iron availability *via* the fragile [4Fe-4S] cluster of the bacterial transcriptional repressor RirA

Maria Teresa Pellicer Martinez, Ana Bermejo Martinez, Jason C. Crack, John D. Holmes, Dimitri A. Svistunenko, Andrew W. B. Johnston, Myles R. Cheesman, Jonathan D. Todd, and Nick E. Le Brun

Supporting Figures

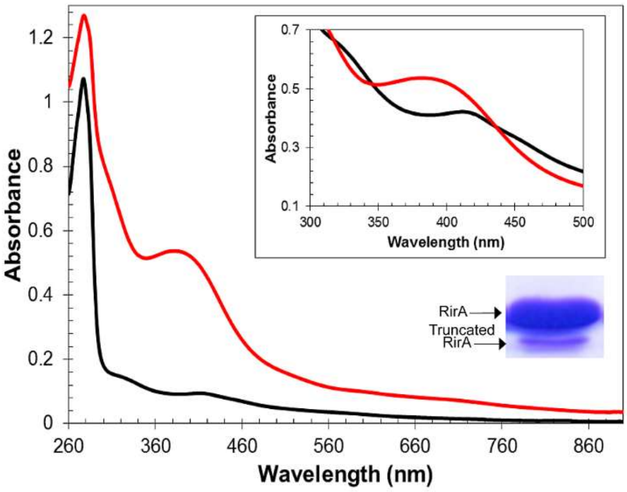

Figure S1. Properties of purified RirA. UV-visible absorption spectra of as isolated (black line) and reconstituted (red line) RirA. Shown in the upper inset is the as isolated spectrum multiplied ×4.5 to permit easier comparison of the shape of the absorbance bands due to the iron-sulfur clusters. Protein samples (as isolated, 1099 μM; reconstituted, 741 μM) were in buffer B (see Materials and methods), pathlength was 1 mm. The lower inset shows an SDS-PAGE of purified as isolated RirA showing the full length and truncated forms of the protein. The truncated form was found to be missing 20 C-terminal residues (M_w 15451 Da) compared to wild type protein (M_w 17441 Da). The significance of truncated RirA is unknown, but its appearance was minimised through optimising the time efficiency of the purification, indicating that it is most likely a post-cell harvest artefact.

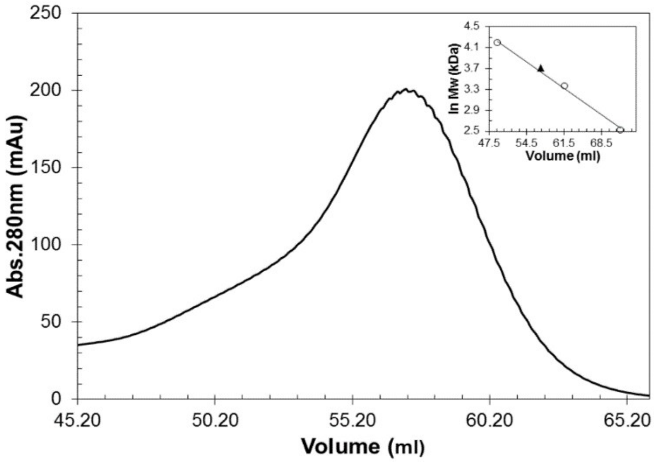

Figure S2. Analysis of RirA association state. Analytical gel filtration chromatogram of RirA (707 µM in [4Fe-4S] cluster). *Inset*, calibration curve for the Sephacryl 100HR column. Standard proteins (open circles) were BSA (66 kDa), carbonic anhydrase (29 kDa), and cytochrome *c* (13 kDa). [4Fe-4S]-RirA is shown as a black triangle. The buffer was 25 mM HEPES, 2.5 mM $CaCl_2$, 50 mM NaCl, 750 mM KCl, pH 7.5. mAu, milli-absorbance units. The observation of one major elution band at a volume consistent with a mass of ~37 kDa indicates that RirA is a dimer. Because the sample was ~57% cluster-loaded, it also suggests that RirA may be a dimer in apo- and cluster-bound forms.

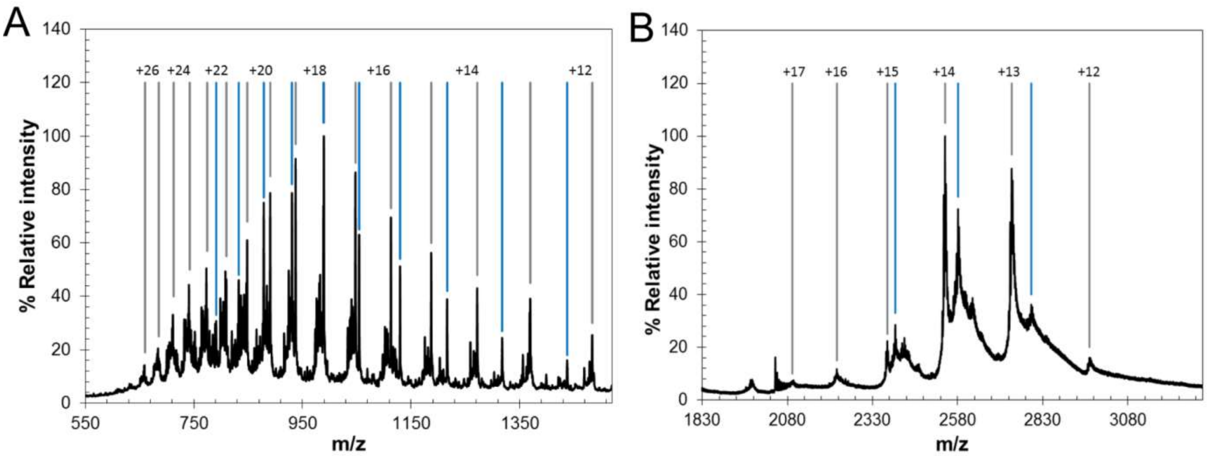

Figure S3. **ESI-MS m/z spectra of [4Fe-4S] RirA**. (**A**) monomer and (**B**) dimer regions of the m/z spectrum are plotted with charge states as indicated. Blue lines indicate the presence of truncated RirA.

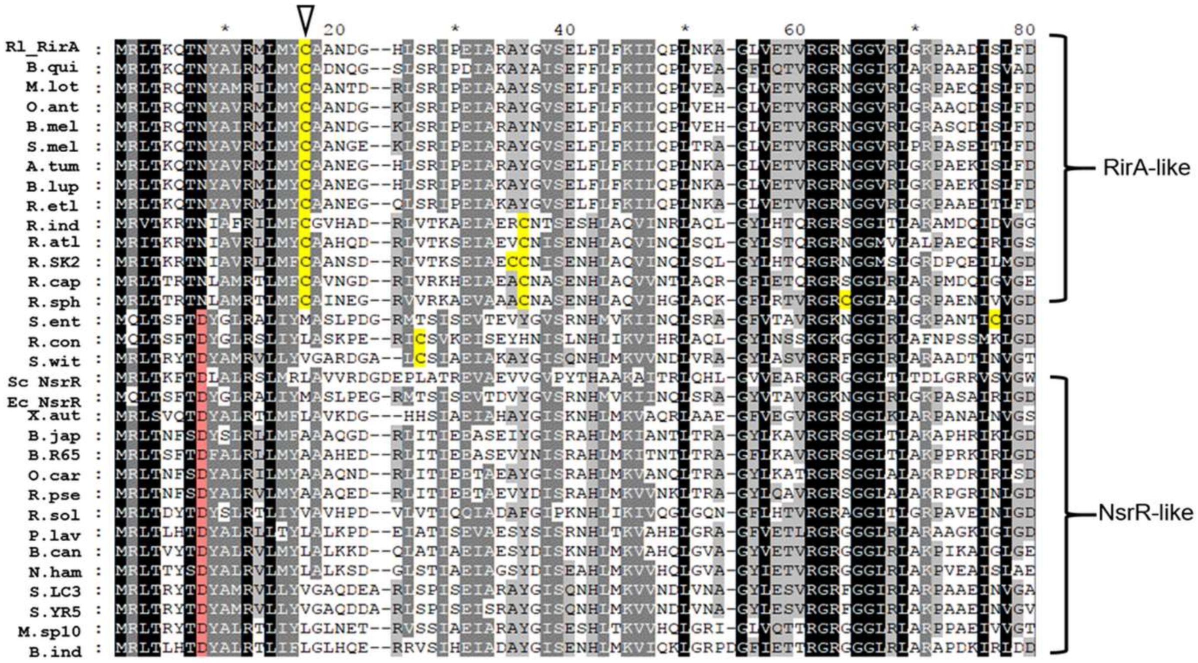

Figure S4. Partial alignment of RirA-like sequences from Rhizobiales, Sphingomonadales and Rhodobacterales. *R. leguminosarum* RirA protein (Rl_RirA) is aligned with homologues from: *Bartonella quintana* (B.qui), *Mesorhizobium loti* (M.lot), *Ochrobactrum anthropic* (O.ant), *Brucella melitensis* (B.mel), *Sinorhizobium meliloti* (S.mel), *Agrobacterium tumefaciens* (A.tum), *Bradyrhizobium lupini* (B.lup), *RhizobiRum etli* (R.etl), *Roseovarius indicus* (R.ind), *Ruegeria atlantica* (R.atl), *Roseobacter sp.* SK209-2-6 (R.SK2), *Rhodobacter capsulatus* (R.cap), *Rhodobacter sphaeroides* (R.sph), *Sphingomonas wittichii* (S.wit), *Xanthobacter autotrophicus* (X.aut), *Bradyrhizobium japonicum* (B.jap), *Bradyrhizobium* sp. ARR65 (B.R65), *Oligotropha carboxidovorans* (O.car), *Rhodopseudomonas pseudopalustris* (R.pse), *Bradyrhizobium canariense* (B.can), *Nitrobacter hamburgensis* (N.ham), *Sphingopyxis sp.* LC363 (S.LC3), *Sphingopyxis sp.* YR583 (S.YR5), *Methylobacterium sp.* 10 (M.sp10), and *Beijerinckia indica* (B.ind). More distantly related sequences from *Salmonella enterica typhimurium* (S.ent), *Rickettsia conorii* str. *Malish7* (R.con), *Ralstonia solanacearum* (R.sol), *Parvibaculum lavamentivorans* (P.lav), *Streptomyces coelicolor* NsrR protein (Sc_NsrR) and *Escherichia coli* NsrR protein (Ec_NsrR) are also included. The sequences can be broadly separated into two distinct groups, corresponding to RirA-like and NsrR-like sequences, as indicated. The three conserved cysteine residues alluded to in the main paper are conserved in all sequences. A fourth cysteine residue that is conserved only in the RirA-like sequences is indicated by an open triangle. Highlighted amino acid residues are as follows: black, totally conserved; grey, semi-conserved; yellow, Cysteine residues; red, residues aligning with Asp8 of *S. coelicolor* NsrR. Proteins were identified by NCBI Blast suite and aligned using Clustal Omega[1] and visualized in Genedoc.[2]

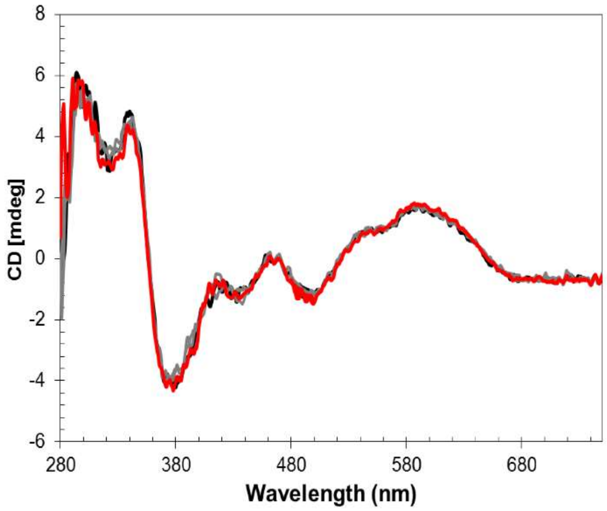

Figure S5. Stability of [4Fe-4S] RirA under anaerobic conditions. CD spectra of 1.4 mM [4Fe-4S] RirA), over a period of 7 hr, in a 1 mm cuvette using buffer B as a baseline. Black and red spectra were recorded at 0 and 7 hr, respectively. Grey spectra were recorded between these time points.

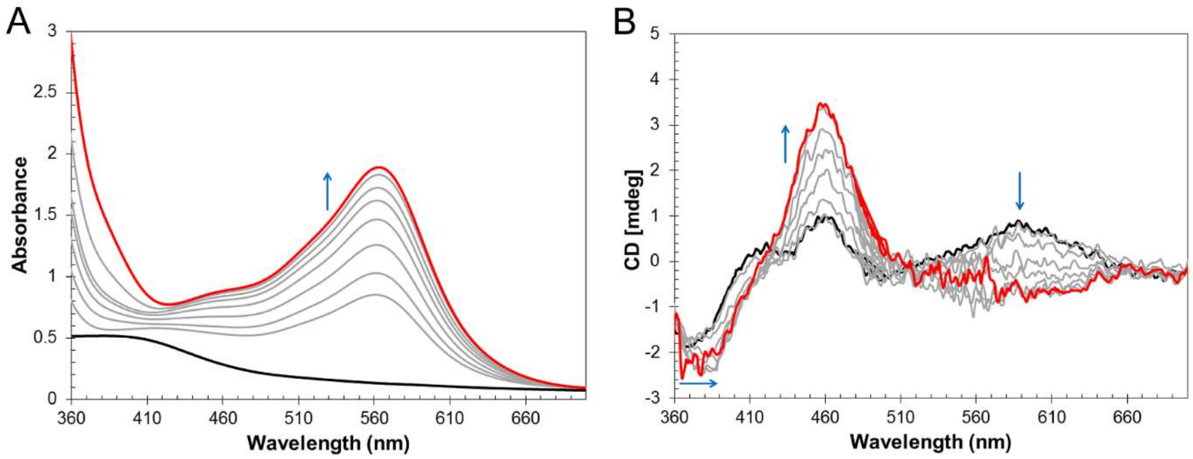

Figure S6. Response of [4Fe-4S] RirA to low iron conditions generated by Ferrozine. (**A**) UV-visible absorbance and (**B**) CD spectra of [4Fe-4S] RirA (37 μM in cluster) following addition of increasing concentrations of the iron chelator Ferrozine (up to 2.3 mM) under anaerobic conditions. Starting and end-point spectra are in black and red, respectively. Arrows show the most significant changes in spectral features during the titration. Pathlength was 1 cm for all measurements. In (A), the changes are due to the formation of the Fe(II)(Ferrozine)$_3$ complex.[3] Using an extinction coefficient at 562 nm of 28,000 M^{-1} cm^{-1}, ~63 μM iron was released during the cluster conversion, equivalent to ~43% of total iron. This is consistent with a [4Fe-4S] to [2Fe-2S] cluster conversion process.

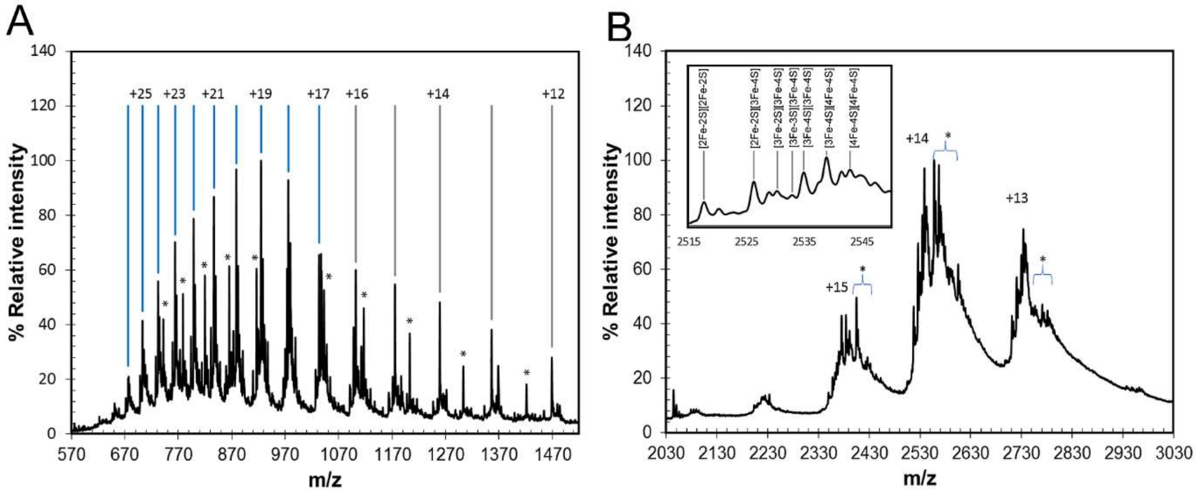

Figure S7. **ESI-MS of EDTA-treated RirA**. m/z spectra of [4Fe-4S] RirA (~21 μM in cluster) in 250 mM ammonium acetate pH 7.35 following exposure to 1 mM EDTA for 2.5 hr and removal of EDTA by gel filtration. (**A**) monomer and (**B**) dimer regions of the m/z spectrum are plotted with charge states as indicated. In (A), black and blue lines indicate the [2Fe-2S] and 2Fe adduct forms of RirA, respectively. In (B), charge states due to cluster-bound forms of dimeric RirA are indicated and the +14 charge state is shown on an expanded m/z scale. Peaks indicated by asterisks are due to overlapping charge states of a truncated form of RirA.

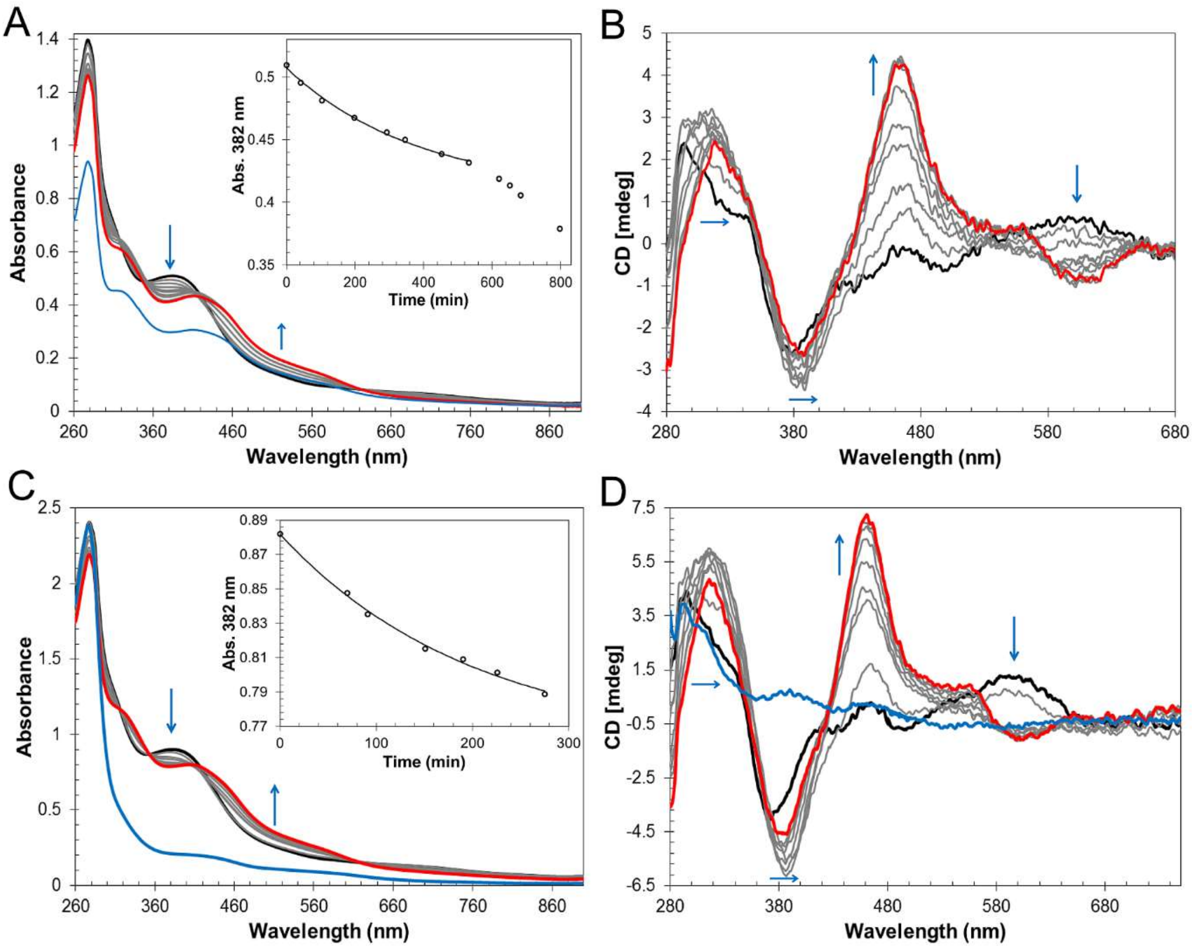

Figure S8. Time dependence of RirA [4Fe-4S] to [2Fe-2S] conversion promoted by different EDTA concentrations under anaerobic conditions. (**A**) UV-visible absorption spectra and (**B**) CD spectra were recorded over several hours following exposure of [4Fe-4S] RirA (28 μM in cluster in buffer B) to 1 mM EDTA under anaerobic conditions. Starting spectrum is in black, end point spectrum is in red and intervening spectra, measured over 17 hr are in grey. In (A) the spectrum of the protein following passage down a gel filtration column equilibrated in buffer B is shown in blue. Inset in (A) is a plot of $A_{382\ nm}$ as a function of time; the solid line represents a fit to the data (k = 2.61 ±0.4 $\times 10^{-3}$ $\times 10^{-3}$ min^{-1}). (**C**) and (**D**), as in (A) and (B), but with [4Fe-4S] RirA (77 μM in cluster in buffer B) following addition of 4 mM EDTA. The spectra in blue were recorded following overnight incubation and centrifugation to remove any precipitated material. Inset in (C) is a plot of $A_{382\ nm}$ as a function of time; the solid line represents a fit of the data (k = 4.96 ±0.79 $\times 10^{-3}$ min^{-1}). Pathlength was 1 cm for all measurements.

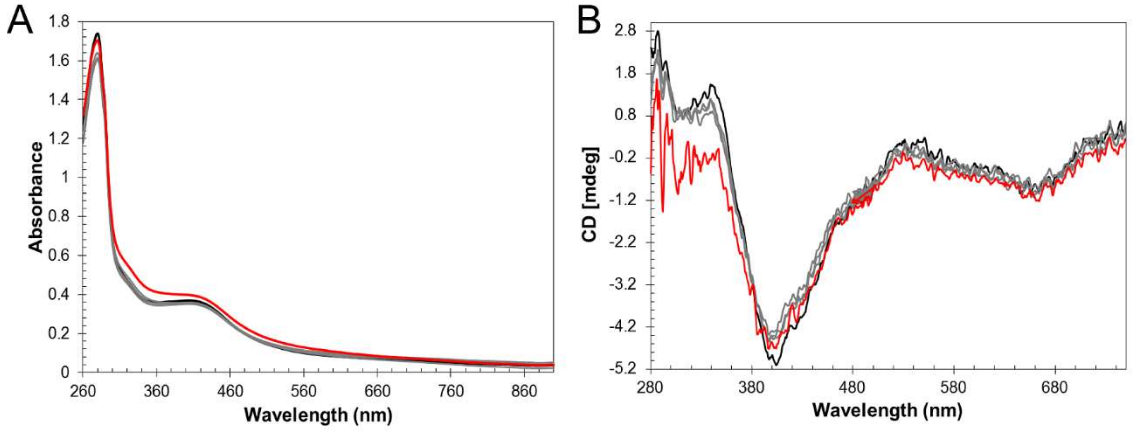

Figure S9. Response of [4Fe-4S] NsrR to low iron conditions generated by Chelex 100. (**A**) UV-visible absorbance and (**B**) CD spectra of [4Fe-4S] NsrR (29 μM in cluster; ~60% cluster-loaded) following addition of Chelex-100 resin separated by a semi-permeable membrane. Starting and end-point spectra are in black and red, respectively. Pathlength was 1 cm for all measurements.

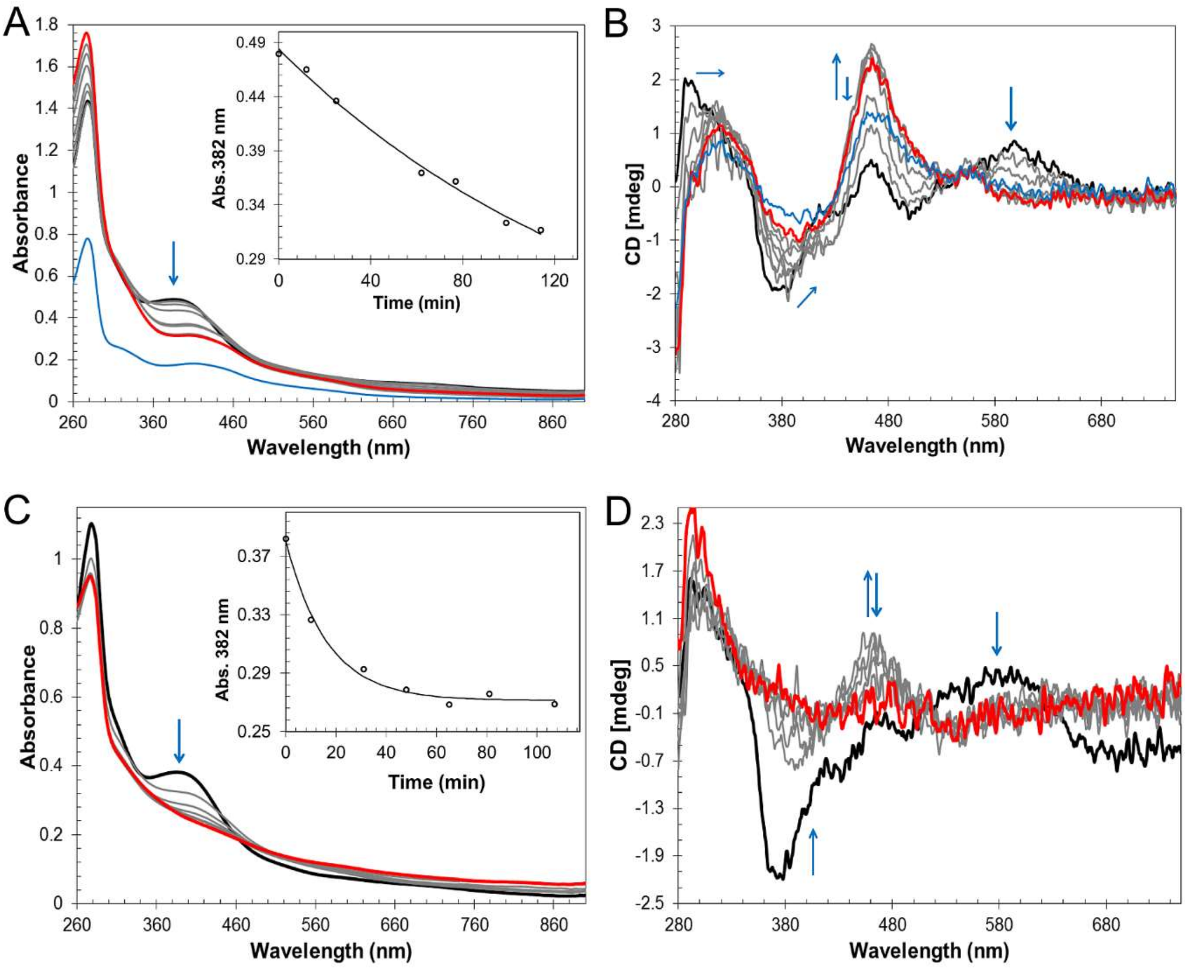

Figure S10. **Time dependence of RirA [4Fe-4S] to [2Fe-2S] conversion promoted by iron chelators under aerobic conditions.** (**A**) UV-visible absorption spectra and (**B**) CD spectra were recorded over several hours following exposure of [4Fe-4S] RirA (28 μM in cluster in buffer B) to 1 mM EDTA under aerobic conditions measured over a period of 2 hr. Starting spectrum is in black, end point spectrum is in red and intervening spectra are in grey. Spectra of the protein following passage down a gel filtration column equilibrated in buffer B are shown in blue. Inset in (A) is a plot of $A_{382\ nm}$ as a function of time; the solid line represents a fit to the data (k = 8.5 ±2.4 ×10^{-3} min^{-1}). (**C**) UV-visible absorption spectra and (**D**) CD spectra were recorded over time following exposure of [4Fe-4S] RirA (30 μM in cluster in buffer B) to Chelex 100 under aerobic conditions. Arrows indicate the most significant spectral changes. Inset in (C) is a plot of $A_{382\ nm}$ as a function of time; the solid line represents a fit of the data (k = 0.061 ±0.007 min^{-1}). Pathlength was 1 cm for all measurements.

5'-CGCAGCCATCGAGGGGGCCCCAGCTGGTCCCGCGGGCTGCCAGGACATCGGCATCTATG
ACCGGACGATCCGGGCTTACCGAGCGACAGGTCTCTTCCCGTAATTGCCGGCGACTCTAATG
GGAGTGGCAGTTGCTCAAGTGAGGGCGATGGTCTTTTGCGCAAGTCCGCCGGCCGACCTATC
CGGCGAGCCACTTCAGCGGCATCTGCAGTCGAAATACCCCTCCTACTCAGTCAGGTGATACT
CTGGCGCGGGCGTGGTTCAAAATCAGCAGCAAATACGGGATCGTCGGGTCCATCCCCGTCAA
GACCGGCAGGTTGGCCATCGTCTCGGTGCGCGCTTGGTCTCGCGCGCGGCAGAACTGCCTCT
GTTTGGCAGCCCCACAGAACGTTACGAGGCCTCGATTCTACGTGCAACCTGTTCGGGAGTGT
TGAAGGCGTGGATTTATCGATTTAAAGGTGACTAAAATAATCATCTTATGTTGACAAGGCCA
ATTTCGCTCCATAGGTTCCGCCCGCATCCGTGGCGTAGGGACCAAACA**ATG**GCACGTGTTTT
TTTGAATGTTTCTAATAATGTATCGCG-3'

Figure S11. Sequence of DNA carrying the *fhuA* promoter. The nucleotides underlined were used as primers to amplify a 581 bp PCR fragment from *Rhizobium leguminosarum* strain J251 genomic DNA, which contains the *fhuA* gene promoter.[2] The *fhuA* start codon is in bold and the IRO sequence is highlighted in grey.

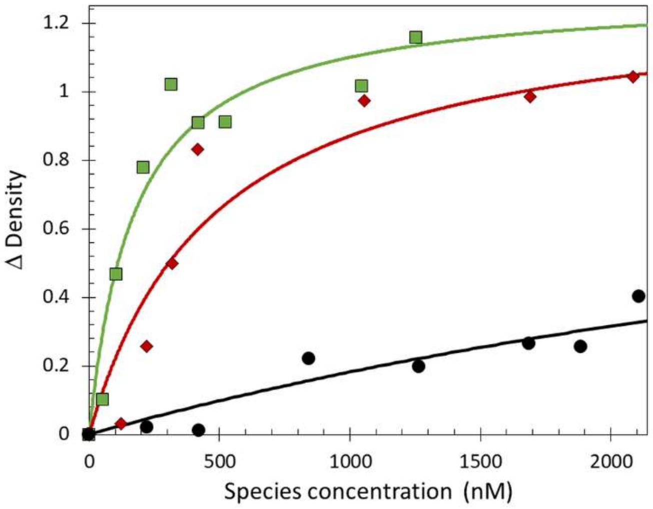

Figure S12. Densitometric analysis of RirA binding to DNA. Densitometric analysis of [4Fe-4S] (green square) [2Fe-2S] (red diamond) and apo- (black circles) RirA species binding to *fhuA* promoter DNA probe. Densitometric analysis of EMSA data shown in Figure 7 fitted using a simple binding equation (solid lines) gave estimates for the K_d for RirA-DNA complexes of 172 (±57) nM, 482 (±97) nM and >5.5 μM for [4Fe-4S], [2Fe-2S] and apo- RirA, respectively.

Supporting references

1. F. Sievers, A. Wilm, D. Dineen, T. J. Gibson, K. Karplus, W. Li, R. Lopez, H. McWilliam, M. Remmert, J. Soding, J. D. Thompson and D. G. Higgins, *Mol Syst Biol*, 2011, **7**, 539.
2. K. B. Nicholas and H. B. J. Nicholas, *Distributed by the authors*, 1997.
3. C. R. Gibbs, *Anal Chem*, 1976, **48**, 1197-1201.
4. K. H. Yeoman, F. Wisniewski-Dye, C. Timony, J. B. Stevens, N. G. deLuca, J. A. Downie and A. W. Johnston, *Microbiology*, 2000, **146**, 829-837.
